# The unseen face of the COVID-19 pandemic

**DOI:** 10.1192/j.eurpsy.2021.1577

**Published:** 2021-08-13

**Authors:** C.E. Anghel, C. Bacila

**Affiliations:** 1 Clinical Departament, The “Dr.Gh.Preda” Psychiatric Hospital of Sibiu, Sibiu, Romania; 2 Faculty Of Medicine, The “Lucian Blaga” University of Sibiu, Sibiu, Romania

**Keywords:** Suicide, pandemic covid-19, inpatients, mental health

## Abstract

**Introduction:**

Starting with December 2019, the first cases of SARS-CoV2 virus appeared in the Wuhan region of China, which will become the COVID-19 pandemic and will have an impact on the bio-psycho-socio-cultural environment. Lockdown and social isolation measures have been imposed in an attempt to gain time and find a viable treatment and a vaccine, for this new infection. The media, in an attempt to promote these measures and information about COVID-19 symptoms, have further increased fear of the virus in population.

**Objectives:**

This presentation tried to observe the impact of the COVID-19 pandemic on patients confirmed positive with SARS- CoV2 infection, treated in hospitals, inpatients who died by suicide.

**Methods:**

As methods a brief review of the literature was made, based on research in scientific articles published in PubMed, APA PsychNet, The BMJ, Who.int, using as keywords the terms “pandemic covid-19”, “inpatients” and “suicide”, published between January 2020 - October 2020.

**Results:**

Several studies conducted to assess the impact of the pandemic on mental health found a significant increase in dysphoria, unhappiness, irritability, anxiety, dominant thoughts related to the transmission of the SARS-CoV2 virus, a tendency to worry about their health and culminating with suicide in the medical unit.

**Conclusions:**

Depending on the psychological structure of each person and the socio-cultural context, different behaviors were observed related to the impact of this pandemic on mental health. The most important is, however, the occurrence of a significant number of deaths by suicide in hospitals in the context of social isolation, patients without a psychiatric history.

